# Considering Sleep, Mood, and Stress in a Family Context: A Preliminary Study

**DOI:** 10.3390/clockssleep1020022

**Published:** 2019-05-10

**Authors:** Valeria Bacaro, Bernd Feige, Andrea Ballesio, Paola De Bartolo, Anna F. Johann, Carlo Buonanno, Francesco Mancini, Caterina Lombardo, Dieter Riemann, Chiara Baglioni

**Affiliations:** 1 Department of Human Sciences, University of Rome “G. Marconi”- Telematic, 00193 Rome, Italy; 2 Department of Psychiatry and Psychotherapy, Medical Center - University of Freiburg, Faculty of Medicine, University of Freiburg, 79110 Freiburg, Germany; 3 Department of Psychology, Sapienza University of Rome, 00185 Rome, Italy; 4 Laboratory of Experimental Neurophysiology, IRCCS Santa Lucia Foundation, 00179 Rome, Italy; 5 Medical Psychology and Medical Sociology, Faculty of Medicine, University of Freiburg, 79110 Freiburg, Germany; 6 Specialization School in Cognitive Psychotherapy S.r.l., Association of Cognitive Psychotherapy, 00185 Rome, Italy

**Keywords:** sleep, infant sleep, family context, stress, sleep habits, mood

## Abstract

Background: During the first years of life, parental sleep strongly depends on child’s sleep quality. Poor parental sleep may relate to increased stress and negative mood. However, there is a lack of sleep studies focusing on all family members. This study aimed to investigate the relationship between sleep, mood, and stress in mothers, fathers and children. Methods: Data were obtained from 65 parental couples and 65 children (2 to 36 months). Data on sleep for all family members and stress of parents were completed by both mothers and fathers through questionnaires and sleep diaries. Results: Toddlers’ positive mood before nocturnal sleep was significantly associated with reduced wake times after sleep onset. Mothers reported worse sleep quality compared to fathers. Shorter sleep onset latency in fathers and better sleep efficiency in mothers were linked with better self-reported mood upon awakening. In mothers, but not in fathers, poor sleep quality was associated with higher perceived stress. Conclusion: Results suggest bidirectional relationships between sleep and mood in children, mothers and fathers. Moreover, results evidence poorer sleep in mothers, compared to fathers, which was linked with increased parenting stress. This gender gap should be further considered in studies with larger samples and in clinical contexts.

## 1. Introduction

The development of regulated and consolidated sleep-wake patterns is one of the most salient developmental processes of early childhood. This process changes rapidly during the first year of life and continues to evolve throughout childhood. Biological rhythms in newborns are not yet established, as their sleep is distributed throughout an entire day in accordance with feeding frequency [1]. Circadian rhythms start occurring at 10/12 weeks of age, when the majority of sleep becomes increasingly nocturnal [2]. Children continue taking daytime naps approximately until their fourth year of life in order to receive the required amount of sleep [3]. Furthermore, frequent night-time awakenings are very common in infancy and early childhood, and the child’s ability to fall back to sleep unassisted plays a major role in determining future sleep problems [4]. 

Parents play a crucial role in the development of regular nocturnal sleep by providing cues preceding sleep (e.g. by implementing consistent feeding times and bed-time routines) during their child’s first years of life [1,5,6,7]. Daycare and school schedules, parenting practices and expectations, family routines and cultural practices are all factors influencing sleep-wake patterns (e.g. sleep duration, sleep onset latency, night-awakenings/self-calming, sleeping arrangements) in infants and children [8]. Infants who fall asleep in their bed without parental assistance compared to infants who fall asleep with significant parental involvement (e.g. while being held, fed, rocked, etc.) are more likely to have an increased number and duration of night-time awakenings [6,9,10]. Using breastfeeding as a strategy to help a child fall asleep is another crucial factor that may influence infant’s sleep. In fact, it has been found that this practice is associated with more frequent night-awakenings and with lower levels of self-soothing [6,9]. 

There is also evidence referring to the relationship between sleep and emotion regulation in children. For example, it has been found that toddlers (aged 30–36 months) with nap restriction were less able to solve a difficult task involving self-regulation strategies compared to toddlers without nap restriction [11]. Moreover, Hysing and colleagues in 2016 [12] found that two year old toddlers who slept less than 11 hours per night and had prolonged sleep onset latency (>30 min) and/or three or more awakenings per night were more likely to develop socio-emotional problems. However, while the associations between sleep and emotional functioning have been extensively studied in later childhood, less is known about these associations in younger children [13]. 

Furthermore, poor sleep in infants and toddlers implies poor sleep of the parents. Mother’s sleep becomes highly fragmented and inefficient in the early postpartum period [14], including higher numbers of night-time awakenings and higher daytime sleepiness compared to what is experienced during pregnancy [15]. More than half (55%) of mothers report poor sleep quality and commonly describe symptoms related to insomnia, as well as requiring longer than 30 min to fall asleep (12.3%), experiencing frequent awakenings during the night or waking up too early in the morning (37.0%) in the postpartum [16]. 

In addition, it has been found that infant’s behavioral sleep problems seem to be associated with maternal stress and poor self-reported health [17], as well as with paternal general health [18] and depressive symptoms [19,20]. Based on this literature, it seems to be essential to better explore the relationships between sleep and mood in children and parents adopting a family perspective. Indeed, most of the studies regarding infant sleep and parenting focus mainly on the assessment of maternal perception and behavior and do not clearly differentiate between maternal and paternal behavior [21].

Therefore, this pilot study is aimed at exploring the relationships between sleep (quality, duration and variability), mood (before and after nocturnal sleep) and perceived stress in mothers and fathers of infants and toddlers (0–36 months). Adopting a family perspective, data on sleep and mood was collected through questionnaires and diaries completed by the parents. We decided to select infants and toddlers in order to focus on the development of sleep patterns before nursery school, where significant changes in sleep and socio-emotional patterns occur. Specifically, we hypothesized that: (1) parental perception of child’s sleep reported at the questionnaires would be associated with child’s sleep quality (i.e. length of sleep onset latency, wake after sleep onset, and total sleep time) as measured with the use of sleep diaries; (2) parents who describe the sleep of their child as problematic would present worse sleep quality and higher perceived stress compared to parents who report no sleep problems in their child, and that this would be emphasized in mothers; (3) independently of parental reported child’s sleep quality, valence of mood before and after sleeping would be associated with sleep quality in all family members; (4) independently of parental reported child’s sleep quality, sleep pattern of parents would be associated with sleep pattern of children and also with perceived stress.

## 2. Results

### 2.1. Sample Characteristics

The sample comprised 65 mothers aged 23–49 years (35.03 ± 4.84 years), 65 fathers aged 24–55 years (38.20 ± 5.95 years) and 65 children (35 females and 30 males) aged 2–36 months (19.16 ± 9.60 months). Forty children were first born, twenty-three were second-born, one was third-born and one was last-born of seven children. All families in the given sample had only one child in the selected age group (see “Method” section). Table 1 shows demographic and co-morbid conditions of the sample. On average mothers reported 15.31 ± 2.93 years of education and fathers 13.06 ± 3.64 years. Two mothers and two fathers reported experiencing current or past (retrospectively estimated) depression while ten mothers and eleven fathers current or past anxiety problems. The results of Insomnia Severity Index (ISI) [22] showed that nine mothers and six fathers suffered from clinical insomnia and twenty-five mothers and twenty-four fathers of subthreshold insomnia. In addition, in the part of the questionnaire related to sociodemographic information parents were asked to retrospectively rate their subjective sleep quality before and after child birth in a scale from 0 = bad to 4 = good. It emerged that both mothers and fathers reported poorer retrospective sleep quality after the child’s birth compared to before the child was born.

Table 2 summarizes descriptive characteristics of sleep patterns, as reported by parents in the questionnaires (in all cases the questionnaires were completed by mothers) of infants and toddlers in our sample divided by age groups. After the first year of life, the duration of sleep in children increases during the night and decreases during the day, the time spent awake during the night is also reduced. However, parents on average reported that, regardless of age, children still require a significant amount of time to fall asleep (>30 min). Forty percent of parents reported putting their children to bed when they were already asleep, while 60% reported allowing children to fall asleep in their own bed. All children were reported to go to bed after 8 p.m., 49% of them were reported to go to bed after 10 p.m. or later. 

### 2.2. Differences between Children with Sleep Problems and Children without Sleep Problems

Based on parental reports (Sleep Disturbance Scale for Children: SDSC [23]; Brief Infant Sleep Questionnaire: BISQ [24]), children were categorized as with and without sleep problems (See “Method” section for full information). The categorization was carried out in accordance with the criteria for pediatric insomnia, including items such as difficulties falling asleep, maintaining sleep during the night and needing parental presence to fall asleep [25,26]. 

The group of children without sleep problems included 20 females and 20 males aged 20.55 ± 8.84 months; the group of children with sleep problems included 15 females and 10 males aged 16.94 ± 10.50 months. As shown in Table 3, the information obtained from sleep diaries was used to describe sleep patterns exhibited by the children with and without sleep problems depending on their age. The sleep diaries for all children in the given sample were completed by mothers (see “Method” section).

Multivariate analysis of variance (MANOVA) was performed to examine parameters obtained from sleep diaries of both groups of children, controlling for the effect of age of children. The results showed that children with sleep problems had significantly longer Wake After Sleep Onset (WASO) (34.53 ± 34.36 min) compared to children without sleep problems (19.38 ± 17.77 min, *F*_(1,59)_ = 13.72, *p* < 0.001, η_p_^2^ = 0.18); no significant result was found for other sleep parameters.

### 2.3. Differences between Mothers and Fathers of Children with or without Sleep Problems on Sleep and Perceived Stress

A 2 × 2 multivariate analysis of variance parent (mother vs. father) * sleep condition (children with/without sleep problems) controlling for the effect of age of children was conducted in order to investigate differences in sleep quality, sleep duration and perceived stress. There was a significant main effect of parent (mothers vs. fathers) on the WASO (*F*_(3,82)_ = 4.83, *p* = 0.03). Mothers, on average, had significant longer WASO (33.71 ± 28.46 min) compared to fathers (23.85 ± 19.23 min). The WASO night-by-night variability was also larger in mothers (28.94 ± 22.14 min vs. 20.04 ± 15.95 min; *F*_(1,82)_ = 4.89, *p* = 0.03). 

Furthermore, there was a significant main effect (*F*_(3,82)_ = 5.84, *p* = 0.01) of sleep condition (children with/without sleep problems) on averaged total sleep time (TST) and the “Parental Distress” subscale of the Parenting Stress Index [27]. Parents of children without sleep problems had significantly higher TST (438.89 ± 53.05 min) and lower scores on the parent distress scale (41.79 ± 9.75) compared to parents of children with sleep problems (402.85 ± 76.09 min and 47.45 ± 10.74 mean score). Finally, the night-by-night variability showed a significant interaction effect between parent group (mothers vs. fathers) and sleep condition (children with/without sleep problems) for TST (*F*_(3,82)_ = 5.63, *p* = 0.02). Specifically, TST variability across the eight days was similar in parents of children with (74.61 ± 38.14 min) and without sleep problems (77.67 ± 43.91 min), but it was higher in fathers (78.89 ± 47.63 min) compared to mothers (74.92 ± 37.51 min).

### 2.4. Relationships between Mood and Sleep in Children and Parents

In order to explore the relationship between mood (before and after nocturnal sleep), sleep quality and sleep duration, partial correlation analysis (one-tail) controlling for age of children and sleep condition (children with/without sleep problems) was conducted between mood (before and after sleep) and sleep variables, independently in children, mothers and fathers. Results on children (Table 4) showed that positive mood before going to bed was associated with reduced WASO (*r* = −0.29, *p* = 0.01), but no association was found with TST and Sleep Onset Latency (SOL). Results on parents revealed that in mothers, positive mood before sleep was associated with reduced WASO (*r* = −0.27, *p* = 0.02) and enhanced Sleep Efficiency (SE) (*r* = 0.38, *p* < 0.005). The results observed in fathers showed that positive mood before going to sleep is associated with shorter SOL (*r* = −0.33, *p* = 0.01).

In addition, partial correlation analysis controlling for the effect of age of children and for the sleep condition showed that in children, longer WASO was associated with worse mood upon awakening (*r* = −0.25, *p* = 0.02). In mothers, higher SE was associated with better mood upon awakening (*r* = 0.29, *p* = 0.01), whereas in fathers longer SOL was associated with worse mood upon awakening (*r* = −0.28, *p* = 0.03). 

### 2.5. Relationships between Sleep Duration and Quality and Perceived Stress in Children and Parents

In order to investigate the relationships between sleep quality and duration in fathers, mothers and children, partial correlation analysis controlling for age of children was performed (see Table 5). Results showed a positive association between SOL of mothers and children (*r* = 0.29, *p* = 0.04). Furthermore, WASO of children was positively associated with WASO of mothers (*r* = 0.79, *p* < 0.001) and fathers (*r* = 0.43, *p* < 0.001). In children, longer WASO was also associated with reduced TST (*r* = −0.43, *p* < 0.005) and reduced SE (*r* = −0.46, *p* < 0.001) of fathers. Finally, total sleep time of children positively correlated with total sleep time of mothers (*r* = 0.31, *p* = 0.03).

Moreover, investigating the relationships between sleep quality, sleep duration and perceived stress has shown that higher reported stress of mothers was associated with reduced TST (*r* = −0.34, *p* = 0.03).

## 3. Discussion

The present study focused on sleep patterns in families and associations between mood and stress in Italian children in early infancy, their mothers and fathers. Infants and toddlers in our sample had on average shorter sleep duration compared to the sleep times recommended by the National Sleep Foundation [28]. Furthermore, infants and toddlers presented long sleep onset latencies (more than 30 min) and longer wake after sleep onset (more than 30 min). The result obtained may be representative of poor knowledge regarding infant’s and children’s sleep in Italy and the fears of Italian parents involving leaving the awake child in their bed unattended, as has been previously documented [29]. Most parents and pediatricians in Italy are unlikely to discourage the excessive parental presence during the night, and usually do not promote proper sleep hygiene from early childhood [30,31].

General poor sleep quality was also reflected in the high number of children with sleep problems (25 out of 65). The main difference between children without sleep problems and children with sleep problems, as described in sleep diaries, was associated with duration of time awake during the night, which was longer in the group with sleep problems. Nevertheless, the groups did not differ in the duration of sleep onset latency, which was found to be actually relatively long throughout the sample. This could be due to the fact that the categorization of children into with and without sleep problems was based on the parents’ perception of children’s sleep problem. Prolonged wakefulness at night may have been perceived as a stressful event by the parents, and especially by mothers (as in our sample, the questionnaires for all children were completed by mothers), compared to prolonged sleep onset latencies. The age of children in our sample ranged from 2 to 36 months, and it is known in literature that in the first three years of life the sleep pattern tends to be very variable e.g., [2]. For this reason, we controlled for the effect of the age of children by including it as a covariate in our analyses e.g., [32].

Upon evaluating both parents with respect to sleep quality and duration, results showed that mothers had worse sleep quality compared to fathers and it was linked to higher levels of stress. Moreover, parents of children with sleep problems reported worse sleep quality, shorter sleep duration and higher levels of stress compared to parents of children without sleep problems. Despite this finding, no significant other differences were found between fathers and mothers of the two groups. Consistently, fathers had, on average, higher sleep efficiency compared to mothers, and this was in accordance with previous results [33,34]. These findings could be explained by the fact that in the majority of families, mothers still have a primary role in taking care of children during the night and this may be strictly linked with high levels of mental and physical fatigue.

Finally, we evaluated the relationships between mood, stress and sleep in children and parents. Prior research has indicated that insomnia is highly correlated with depression and anxiety [35] as well as with an increased risk of developing mood disorders [36]. Nevertheless, little is known about the relationships between non-pathological mood states and sleep. In their review, Baglioni et al. [32] concluded that positive affect may predict reduced sleep difficulty, such as shorter sleep onset latency and longer total sleep time [37,38,39]. Conversely, greater negative affect predicts longer sleep onset latency and shorter sleep duration [40,41]. Based on these hypotheses, our study investigated the relationships between mood (before and after nocturnal sleep) in parents and children and sleep duration and quality. Results showed that there was a reciprocal relationship between mood and wakefulness after sleep onset in children. This finding suggests that, in toddlers, mood before going to bed could influence nocturnal sleep, and that nocturnal sleep of good quality could influence mood in the morning. Moreover, we found a significant negative correlation between sleep onset latency and positive mood after nocturnal sleep in fathers, but not in mothers, and a reciprocal relationship between mood and sleep efficiency in mothers. It is known in the literature that sleep quality is positively associated with a subsequent change in positive affect, and negatively associated with a subsequent change in negative affect [42].

Preliminary results were found regarding the relationships between sleep duration and quality and perceived stress in parents. In fact, results showed a significant correlation between scores in “Parental Distress” subscale of the Parenting Stress Index and total sleep time of mothers, but no other significant results were found. Future studies should include questions about perceived stress before going to bed in order to have more accurate measures of perceived stress during the day and in order to investigate the association with nocturnal sleep.

This study has several limitations, as initially a relatively small sample was recruited and this could impact the generalizability of the results. Additionally, in the study, only subjective and self-reported measures were used and this could create a bias in the results. It would be interesting for future research to use physiological and objective measures such as actigraphy or polysomnography to assess sleep patterns. Another limitation of this study is that the mood assessment was carried out exclusively with the use of diaries. In future studies it could be useful to add more detailed questions about mood before and after sleep, and additionally to assess arousal with certain physiological measures, e.g. skin conductance and heart rate. Furthermore, despite having a general measure of self-reported perceived stress, it could be interesting to assess this before going to bed in order to obtain a rate of the perceived stress during the day and to analyze how it influences the subsequent sleep. Finally, this study is a preliminary study, in which descriptive and exploratory analyses were performed, which decreases the impact of findings. Future studies would involve performing more sophisticated statistical analyses, to assess, for example, the causality of positive or bad mood and better/worse sleep quality.

## 4. Materials and Methods

### 4.1. Participants and Procedure

Following the inclusion criteria, participants were recruited within Italian families with at least one child aged between 0 to 36 months, without acute or relevant physical or psychological pathologies. Psychology students of University of Rome Guglielmo Marconi recruited the participants among families and acquaintances, through advertising in schools, nurseries and workplaces as a part of their practical curricular exercise.

Families potentially interested in participation were given a detailed explanation of the study procedure at the first meeting and were asked to sign an informed consent sheet. Firstly, mothers and fathers were asked to complete a battery of questionnaires (see below). Subsequently, each family was provided with an eight-day sleep diary, and participants were instructed to complete the diary within 30 min after final awakening in the morning. They were also asked to follow the same instructions when completing the child’s sleep diary. Every morning students contacted families to resolve any doubts or problems that may have occurred during the completion of diaries. At the end of the eight days, participants returned the material. 

All procedures were performed in accordance with the 1964 Helsinki Declaration and its later amendments, and it was approved by the local ethical committee of the Department of Educational Sciences of the University of Rome Guglielmo Marconi.

### 4.2. Instruments

#### 4.2.1. Parenting Stress Index- Short Form (PSI-SF)

The Parenting Stress Index–Short Form is a self-report measure that assesses parenting stress in parents of children aged 3 months to 10 years. The PSI Short Form is comprised of 36 items divided in three sub-scales: “Parental Distress”, “Parent–Child Dysfunctional Interaction”, and “Difficult Child Characteristics”. Parents rated their agreement with each questionnaire item on a five-point Likert scale from 1 (Strongly Agree) to 5 (Strongly Disagree) [27]. 

#### 4.2.2. Insomnia Severity Index (ISI)

The Italian version of the ISI [22,43,44] was used to obtain a parametric measure of insomnia severity in parents. Answers were provided on a five-point Likert scale, and summing up the results of the respective seven items, ranging from 0 to 28, a total score of insomnia severity during the preceding two weeks could be obtained. The total score is interpreted as follows: clinically irrelevant insomnia (0–7); subthreshold insomnia (8–14); moderate insomnia (15–21); and severe insomnia (22–28). 

#### 4.2.3. Questionnaire Concerning Sociodemographic Information

Parents were asked to complete a specifically created questionnaire that investigated age, work position and work satisfaction (from 0 = at all, to 4 = a lot), retrospective sleep quality before and after the birth of the child (from 0 = bad to 4 = good), and family organization (presence of baby sitter, grandparents and level of satisfaction concerning the issue). Additionally, information about present and past psychopathologies and insomnia were requested.

#### 4.2.4. Sleep Disturbance Scale for Children (SDSC)

This questionnaire is composed of three parts. The first part assessed the medical history of the child from birth. The second part included questions concerning child’s sleep habits and sleep disorders. In order to investigate the presence of sleep disorders, parents were asked to answer questions such as “Does your child take more than 30 min to fall asleep?”; “Does your child wake up more than two times during the night?”, with three possible answers: 0 = 1 time a week, 1 = 2–4 times a week, and 2 = 5 or more times a week. The last part assessed the presence of sleep disorders in the family, investigating history and type of sleep disorders in the family members. All questions referred to the past 6 months of the child’s life [23]. 

#### 4.2.5. Brief Infant Sleep Questionnaire (BISQ)

The BISQ is a brief, nine-item, multiple choice scale. This questionnaire included specific questions regarding habitual sleep pattern, e.g., the amount of diurnal and nocturnal sleep, sleep onset latency and wake after sleep onset. Additionally, parents were asked to answer questions concerning sleeping arrangements, bedtime rituals and other parental interventions. The scale included questions such as “How does your child fall asleep?”, where the possible answers were: “alone in his/her bed”, “during breastfeeding”, “in bed with parents”, “while he/she is being cradled” [24].

#### 4.2.6. Sleep Diary

Infants’ and parents’ sleep was assessed by sleep diaries for eight consecutive days. The parents’ diary was consistent with the consensus sleep diary [45]. Each morning, mothers and fathers were asked to rate their mood before going to bed on a scale ranging from 0 = very bad to 4 = very good; time of going to bed; time of falling asleep; sleep onset latency; number and duration of nocturnal awakenings; time of waking up and time of getting out of bed. The parents were also asked to rate their mood at the awakening time from 0 = very bad to 4 = very good. 

Additionally, parents were instructed to complete an eight-day sleep diary for their child. Parents were instructed to report child’s time of going to bed; if the child was awake or already sleeping at that time; mood of the child before going to bed from 0 = very bad to 4 = very good; time of falling asleep; sleep onset latency; any infant night waking of which they were aware and its length; waking up time and child’s mood at the awakening time from 0 = very bad to 4 = very good. 

From the information reported in diaries, it is possible to derive total sleep time (TST), sleep onset latency (SOL) and wakefulness after sleep onset (WASO) of parents and children as well as total time in bed (TBT) and sleep efficiency (SE, as TST/TBT*100) of parents. The sleep diary is considered the gold standard among subjective measures of sleep [46]. 

### 4.3. Statistical Analysis

Using the data extracted from sleep diaries of parents and children, the following sleep indices were calculated: SOL, WASO, TST, TBT, and SE. Variability in sleep parameters was calculated as the mean absolute consecutive difference. As an example for three nights, if SOL was 30, 20 and 10 min, sleep variability was calculated as 30–20 = 10, 20–10 = 10, with a mean variability of 10 min. Sleep parameters and mood before and after nocturnal sleep of parents and children were averaged over the eight nights of the study and then analyzed. Data of all 65 families could be included because there were at most three unanswered questionnaire items per family, and these were all items irrelevant for calculating sleep parameters or scoring.

All statistical analyses were performed using SPSS software, version 25.0 (SPSS Inc, Chicago, IL). Descriptive and frequency analyses on sociodemographic questionnaire and on BISQ [24] were performed to analyze sample’s characteristics, children’s habitual sleep pattern and parental involvement. Based on the information acquired from BISQ [24] and SDSC [23], where parents answered questions concerning their child’s sleep habits and problems, and following pediatric insomnia criteria [25,26], the sample was split into two different groups of children: children with sleep problems and children without sleep problems. Specifically, the criteria for children with sleep problems were:

- *Sleep onset protodyssomnia (SOP)* was defined by sleep onset latency of more than 30 min for five or more times per week (parents had to answer “5 or more times a week” in item: “Your child takes more than 30 min to fall asleep” of the SDSC ) and the presence of one of the parents to fall asleep (parents had to report that the presence of one of the parents was required for the child to fall asleep in item: “How does your child fall asleep?” of the BISQ).
- *Night waking protodyssomnia (NWP)* was defined by two or more awakenings during the night for five or more times per week (Parents had to answer “5 or more times a week” in item: "Your child wakes up more than 2 times during the night” of the BISQ).
- *Mixed insomnia* (Mixed I) was defined by presence of both SOP and NWP.
- *Shortened Total Sleep time* (STS) was defined by total sleep duration of the child in 24 hours span reported as below the sleep duration recommended by the National Sleep Foundation [28].

The sample included four children with NWP, 10 children with SOP, seven children with Mixed I, four children with STS and 40 good sleepers. On this basis a division into two groups consisting of 40 children without sleep problems and 25 children with sleep problems was carried out. Before proceeding with the analyses, we checked for the assumptions using SPSS software (P-P plot; skewness, kurtosis and Levene’s test). 

Descriptive analyses were performed in order to investigate differences in two groups of children and diary-based sleep patterns (TST, SOL and WASO), for children’s group and age. Moreover, multivariate analysis of variance using age of children as covariate (MANCOVA) was conducted in order to investigate significant differences in diary-based sleep patterns between the two groups of children.

Multivariate factorial analysis of variance 2 × 2 parent (mother vs. father) * sleep condition (Children with/without sleep problems) was performed in order to investigate differences in sleep quality and duration (using sleep parameters acquired from sleep diaries) between mothers and fathers of children with and without sleep problems, controlling for the effect of age of children. Furthermore, in order to explore the relationships between mood (before and after nocturnal sleep), quality and duration of sleep and perceived stress in parents and children, partial correlations analyses controlling for the effect of age of children and the sleep condition (children with or without sleep problems) were performed independently in children and parents.

## 5. Conclusions

These preliminary results suggest a close link between mood and stress on one side and sleep quality on the other side in all family members (e.g., child, mother, and father). Longitudinal studies should be conducted to clarify whether this link is bidirectional or one variable is predictive of the other. As expected, child’s sleep patterns influence parents’, and particularly mothers’, sleep patterns. Differences in psychophysiological health parameters between mothers and fathers of infants and toddlers have been poorly investigated. There is an utmost need of increasing research on maternal’s health to guide new clinical paradigms. 

## Figures and Tables

**Table 1:** Sample characteristic.

			*Mothers (N = 65)*	*Fathers (N = 65)*	*Children (N = 65)*
*Demographics*			*(Mean ± SD)*	*(Mean ± SD)*	*(Mean ± SD)*
	Age		35.03 ± 4.84	38.20 ± 5.95	19.6 ± 9.60
	Education level		15.31 ± 2.93	13.06 ± 3.64	
*Co-morbid conditions*			*N=*	*N=*	
	Depression		2	2	
	Anxiety		10	11	
	Subthresold insomnia		25	24	
	Clinical insomnia		9	6	
*Retrospective sleep quality*			*(Mean ± SD)*	*(Mean ± SD)*	
	Before child’s birth		3.48 ± 0.86	3.40 ± 0.82	
	After child’s birth		1.72 ± 1.03	2.17 ± 1.08	

(Age of parents = in years; age of children = in months; education level = years of education; co-morbid conditions = depression and anxiety = self-reported in sociodemographic questionnaire; subthresold and clinical insomnia = from Insomnia Severity Index scores; retrospective sleep quality = retrospectively self-reported in sociodemographic questionnaire from 0 = bad to 4 = good).

**Table 2:** Parental report of children’s sleep pattern.

Children’s Age Group	How Much Time Does Your Child Spend Sleeping During the Night? (In Hours)	How Much Time Does Your Child Spend Sleeping During the Day (In Hours)	How Long Does It Take to Let Your Child Fall Asleep in the Evening? (In Minutes)	How Much Time Does Your Child Spend Awake During The Night? (In Minutes)
0–12 months (*N* = 18)	8.08 ± 1.35	3.60 ± 1.89	30.67 ± 32.20	37.03 ± 56.81
13–24 months (*N* = 27)	8.98 ± 1.10	1.78 ± 0.80	37.04 ± 55.35	18.74 ± 22.65
25–36 months (*N* = 20)	8.94 ± 1.21	1.64 ± 1.06	36.00 ± 29.71	13.85 ± 14.40

**Table 3:** Sleep diaries data of children and toddlers with and without sleep problems.

	Children without Sleep Problems (*N* = 40)				Children with Sleep Problems (*N* = 25)			
	0–12 Months (*N* = 7)	13–24 Months (*N* = 19)	25–36 Months (*N* = 14)	Overall	0–12 Months (*N* = 11)	13–24 Months (*N* = 8)	25–36 Months (*N* = 6)	Overall
**Total sleep time (min)**	572.24 ± 53.90	586.63 ± 52.45	519.78 ± 14.71	561.77 ± 94.58	515.93 ± 104.62	548.10 ± 57.46	530.20 ± 37.91	529.65 ± 77.61
**Sleep onset latency (min)**	9.27 ± 10.15	18.30 ± 12.06	23.27 ± 21.79	18.46 ± 16.27	10.37 ± 10.54	18.08 ± 11.02	23.12 ± 18.55	16.38 ± 13.62
**Wake after sleep onset (min)**	36.66 ± 28.54	17.89 ± 13.70	12.77 ± 8.72	19.38 ± 17.54	49.90 ± 36.71	33.96 ± 36.43	11.97 ± 12.07	35.70 ± 34.83

**Table 4:** Age-corrected partial correlations between pre-bed mood of children and sleep diaries’ pattern.

	Pre-Bed Mood	SOL	TST	WASO
Pre-bed mood	1.00	−0.21 *	0.04	−0.29 **
SOL	−0.21 *	1.00	−0.39 **	−0.00
TST	0.04	−0.39 **	1.00	−0.28 **
WASO	−0.29 **	−0.00	−0.28 **	1.00

Note: SOL = sleep onset latency; TST = total sleep time; WASO = wake after sleep onset. * Significant values at *p* < 0.05, ** Significant values at *p* < 0.01, df = 57.

**Table 5:** Age-corrected partial correlations between sleep diaries’ pattern of fathers, mothers and children.

	SOL of Fathers	SOL of Mothers	SOL of Children	WASO of Fathers	WASO of Mothers	WASO of Children	TST of Fathers	TST of Mothers	TST of Children	SEI of Fathers	SEI of Mothers
SOL of fathers	1.00	0.38	−0.08	0.01	0.30	0.03	−0.11	−0.03	0.28	−0.35 **	−0.40 **
SOL of mothers	0.38 **	1.00	0.29 *	−0.13	−0.07	−0.14	0.00	0.19	0.05	0.04	−0.17
SOL of children	−0.08	0.29 *	1.00	−0.20	0.04	0.16	0.10	0.00	−0.47 **	0.22	0.02
WASO of fathers	0.18	−0.13	−0.20	1.00	0.47 **	0.43 **	−0.48 **	−0.11	0.22	−0.62 **	−0.13
WASO of mothers	0.30 *	−0.07	0.04	0.47 **	1.00	0.79 **	−0.35 **	−0.22	−0.07	−0.51 **	−0.52 **
WASO of children	0.03	−0.14	0.16	0.43 **	0.79 **	1.00	−0.43 **	−0.18	−0.16	−0.46	−0.22
TST of fathers	−0.11	0.00	0.10	−0.48 **	−0.35 **	−0.43 **	1.00	0.08	−0.08	0.57 **	−0.03
TST of mothers	−0.02	0.19	−0.00	−0.12	−0.23	−0.18	0.08	1.00	0.31 *	0.11	0.22
TST of children	0.28 *	0.05	−0.47 **	0.22	−0.07	−0.16	−0.08	0.31 *	1.00	−0.22	−0.03
SEI of fathers	−0.35 **	0.04	0.22	−0.62 **	−0.51 **	−0.46 **	0.57 **	0.11	−0.22	1.00	0.32 *
SEI of mothers	−0.40 **	−0.17	0.02	−0.13	−0.52 **	−0.22	−0.03	0.22	−0.03	0.32 *	1.00

Note: SEI = sleep efficiency index; SOL = sleep onset latency; TST = total sleep time; WASO = wake after sleep onset. * Significant values at *p* < 0.05, ** Significant values at *p* < 0.01, df = 37.

## Abbreviations

ISI	Insomnia Severity Index
BISQ	Brief Infant Sleep Questionnaire
SDSC	Sleep Disturbance Scale for Children
SOP	Sleep Onset Protodyssomnia
SNW	Sleep Maintenance Protodyssomnia
STS	Short Total Sleep
TST	Total Sleep Time
TBT	Total Bed Time
SE	Sleep Efficiency
SOL	Sleep onset latency
WASO	Wake after sleep onset
